# Hail and Farewell

**DOI:** 10.1111/anec.13055

**Published:** 2023-03-17

**Authors:** Mark Haigney

**Affiliations:** ^1^ Hebert School of Medicine Bethesda MD USA

It is a great honor to take over as Editor‐in‐Chief of the Annals of Noninvasive Electrocardiology from my longtime friend, ISHNE colleague, and collaborator Wojciech Zareba, MD, PhD. Having inherited the editorship from Arthur Moss, Wojciech has worked tirelessly for 11 years to strengthen the Annals. In this time, Wojciech has successfully delivered this journal through a period of great upheaval in publishing, leaving it stronger than ever. In 2021 alone, downloads increased by over 35%, outstripping Wiley's other cardiovascular journals which grew by 21.5%. This spectacular record reflects the quality of the manuscripts that were submitted, but ultimately the person in charge must accept the blame for failure or, in this case, the credit for success. Wojciech accomplished this while continuing research activities at the University of Rochester, serving as Principal Investigator in numerous NIH and industry funded trials, and publishing at a furious pace. This September, I had the pleasure of seeing him installed as the inaugural David Mortara Endowed Professor of Cardiology at the University of Rochester, the highest academic honor that can be bestowed. His will be a tough act to follow.

No less important to the success of the Annals has been Grazyna Zareba, PhD, who has acted as Managing Editor for over a decade. Grazyna has been responsible for shepherding every manuscript through the stages of publication, and the readers of the Annals owe her a debt of gratitude.

I look forward to working closely with three superb associate editors. Yi‐Gang Li, MD, is professor of medicine and Director of Cardiology at Xinhua Hospital, School of Medicine, Shanghai Jiao Tong University. Dr. Li is an electrophysiologist who serves as Vice President of the Chinese Heart Rhythm Society, and is a very busy and productive investigator in the treatment of atrial fibrillation and other rhythm disorders.

Craig Dobson, MD, is the Director, Cardiac Clinical Operations, University of Pittsburgh Children's Hospital, in Pittsburgh, Pennsylvania. We have worked closely for 15 years. Craig is an expert in inherited cardiac diseases, and as a pediatric cardiologist brings a unique perspective. Noninvasive techniques for the diagnosis and follow up of congenital heart disease are pivotal in pediatric cardiology, and I believe the Annals will benefit from his experience, expertise, and perspective. In addition, Dr. Dobson is a retired US Army officer who has seen service in Afghanistan and Iraq, where he practiced medicine under the worst conceivable circumstances.

Mori Krantz, MD, is also a non‐electrophysiologist who nevertheless has spent tremendous energy improving our understanding of the impact of drugs and disease on the surface electrocardiogram. As a professor of medicine at the University of Colorado in Denver, Colorado, Mori has dedicated himself to the use of electrocardiography to prevent sudden death in traditionally underserved populations such as those with anorexia nervosa and opioid use disorder. He advises the US Food and Drug Administration and other agencies charged with protecting consumers from off‐target effects of new pharmaceuticals. Mori and I have worked together for 15 years, and I am delighted he will be adding his expertise to Annals.

I am grateful to the Board of Trustees of the International Society for Holter and Noninvasive Electrocardiology for their confidence in me as I take on this new adventure. The Annals is the official journal of ISHNE, the society founded by cardiology legends Harold Kennedy, Peter Cohn, Dan Tzivoni, Arthur Moss, Antoni Bayés de Luna, Vincentz Hombach, Shlomo Stern, and Heinz Weber. My heartfelt thanks go to ISHNE president Takanori Ikeda and president‐elect Vassilios Vassilikos. I am truly standing on the shoulders of giants.

Finally, I look forward to working with our publishers, John Wiley & Sons, Inc. (known as Wiley). The publication of the Annals depends on an international collaboration of authors, editors, and publishers.

Please join me in congratulating our outgoing editorial team, Wojciech and Grazyna Zareba!

